# Correction: Dosage compensation can buffer copy-number variation in wild yeast

**DOI:** 10.7554/eLife.15743

**Published:** 2016-03-23

**Authors:** James Hose, Chris Mun Yong, Maria Sardi, Zhishi Wang, Michael A Newton, Audrey P Gasch

Hose J, Yong CM, Sardi M, Wang Z, Newton MA, Gasch AP. 2015. Dosage compensation can buffer copy-number variation in wild yeast. *eLife* **4**:e05462. doi: 10.7554/eLife.05462.

Published 8 May 2015

We detected an error in the published abstract, where '>30%' should read '10–30%'. The correction matches the values cited throughout the manuscript text, which report that 10–30% of amplified genes, dependent on yeast strain and affected chromosome, display an expression phenotype consistent with dosage compensation.

The article has been corrected accordingly.

